# Evaluating and optimizing the operation of the hydropower system in the Upper Yellow River: A general LINGO-based integrated framework

**DOI:** 10.1371/journal.pone.0191483

**Published:** 2018-01-25

**Authors:** Yuan Si, Xiang Li, Dongqin Yin, Ronghua Liu, Jiahua Wei, Yuefei Huang, Tiejian Li, Jiahong Liu, Shenglong Gu, Guangqian Wang

**Affiliations:** 1 State Key Laboratory of Hydro-Science and Engineering, Tsinghua University, Beijing, China; 2 State Key Laboratory of Simulation and Regulation of Water Cycle in River Basin, China Institute of Water Resources and Hydropower Research, Beijing, China; 3 State Key Laboratory of Plateau Ecology and Agriculture, Qinghai University, Xining, China; 4 Electric Power Planning and Engineering Institute, Beijing, China; Universita degli Studi della Tuscia, ITALY

## Abstract

The hydropower system in the Upper Yellow River (UYR), one of the largest hydropower bases in China, plays a vital role in the energy structure of the Qinghai Power Grid. Due to management difficulties, there is still considerable room for improvement in the joint operation of this system. This paper presents a general LINGO-based integrated framework to study the operation of the UYR hydropower system. The framework is easy to use for operators with little experience in mathematical modeling, takes full advantage of LINGO’s capabilities (such as its solving capacity and multi-threading ability), and packs its three layers (the user layer, the coordination layer, and the base layer) together into an integrated solution that is robust and efficient and represents an effective tool for data/scenario management and analysis. The framework is general and can be easily transferred to other hydropower systems with minimal effort, and it can be extended as the base layer is enriched. The multi-objective model that represents the trade-off between power quantity (i.e., maximum energy production) and power reliability (i.e., firm output) of hydropower operation has been formulated. With equivalent transformations, the optimization problem can be solved by the nonlinear programming (NLP) solvers embedded in the LINGO software, such as the General Solver, the Multi-start Solver, and the Global Solver. Both simulation and optimization are performed to verify the model’s accuracy and to evaluate the operation of the UYR hydropower system. A total of 13 hydropower plants currently in operation are involved, including two pivotal storage reservoirs on the Yellow River, which are the Longyangxia Reservoir and the Liujiaxia Reservoir. Historical hydrological data from multiple years (2000–2010) are provided as input to the model for analysis. The results are as follows. 1) Assuming that the reservoirs are all in operation (in fact, some reservoirs were not operational or did not collect all of the relevant data during the study period), the energy production is estimated as 267.7, 357.5, and 358.3×10^8^ KWh for the Qinghai Power Grid during dry, normal, and wet years, respectively. 2) Assuming that the hydropower system is operated jointly, the firm output can reach 3110 MW (reliability of 100%) and 3510 MW (reliability of 90%). Moreover, a decrease in energy production from the Longyangxia Reservoir can bring about a very large increase in firm output from the hydropower system. 3) The maximum energy production can reach 297.7, 363.9, and 411.4×10^8^ KWh during dry, normal, and wet years, respectively. The trade-off curve between maximum energy production and firm output is also provided for reference.

## Introduction

The characteristics of low cost, limited outputs of pollution and rapid start-up/shut-down make hydropower one of the most promising renewable energy resources. The construction of cascaded hydropower systems has grown rapidly in China in order to make full use of the hydropower resources within river basins. Throughout the nation, 13 large-scale hydropower bases are planned to be constructed along major rivers [1, 2]. The hydropower system in the Upper Yellow River (UYR) is one of the largest hydropower bases. According to a report from the Huanghe Hydropower Development Co., Ltd. [3], a total of 39 hydropower plants will be built or have been built along the main stream of the UYR, and these plants will have a total installed capacity of approximately 25,000 MW. By the end of 2015, 24 power plants had been put into operation, and the designed annual power generation can reach approximately 538×10^8^ KWh, resulting from a total water head of over 2500 m. The UYR hydropower system plays strategic roles in conserving water resources and utilizing hydropower resources. It also serves other purposes for the lower basin area, such as ice/flood control, water supply, and ecological flow.

For quite a long time, the Yellow River has been thought to be an unmanageable river, due to the disharmonious relationships between the scarce water resources and dense population in the surrounding area and the wide expanses of cultivated land, as well as the occurrence of severe sediment deposition [4]. The frequent zero-flow events in the last century threatened the survival of human beings and socio-economic development in the areas along the Yellow River. This situation has been mitigated since 1999, when the unified water flow regulation policy was launched by the Yellow River Conservancy Commission (YRCC). Moreover, the newly released “13th Five-Year Plan for Hydropower” notes that, from now on, studies should focus on management once reservoirs have been built up and put into operation [5]. These policies and regulations provide important basis for enabling the joint operation of a hydropower system such as that of the UYR in the future and closing the gap between theory and practice.

At present, the power balance situation of the Qinghai Power Grid, where the majority of installed capacity of the UYR hydropower system is located, is unfavorable. From the perspectives of both demand and supply, it is estimated that the power consumption throughout Qinghai Province will increase from 911×10^8^ KWh in 2015 to 1342×10^8^ KWh in 2020, whereas the power supply will increase from 540×10^8^ KWh in 2015 to 1067×10^8^ KWh in 2020 [6]. The power shortage is either severe currently or will become severe in the near future. The reasons underlying this power shortage are as follows. 1) Industry, which is the major component of power consumption, has experienced rapid development in recent years. 2) The construction of hydro- and thermal power plants progresses relatively slowly. The power shortage can be offset by both purchasing power from outside the province and making full use of power sources within the province. Hydropower is the most important component of the energy structure of the Qinghai Power Grid, and it generates more than 60% of the power supplied to the province. Therefore, speeding up the construction of the planned hydropower plants and improving the operation of the current hydropower system are keys for relieving power supply pressure on the provincial grid.

Over the last two decades, a series of studies have been conducted on benefit evaluations and comparisons between the individual and joint operation of reservoirs in a river basin. The results indicate that enormous socio-economic benefits can be gained from coordinated operation of reservoirs that have hydraulic, hydrological, and electrical connections [7–11]. Generally, reservoir operation involves multiple conflicting objectives. For decision makers, the Pareto set of solutions for multi-objective reservoir operation is more useful for making decisions than a single solution. The commonly-used method for solving multi-objective optimization problems involves using the traditional mathematical programming methods (such as linear programming [12], successive linear programming [13], generalized reduced gradient algorithm [14], dynamic programming [15], etc.) with weighting or epsilon constraint methods and finding the Pareto set of solutions (i.e., the non-dominated solutions) using a number runs, as described in [16–18]. An alternative method involves implementing evolutionary optimization algorithms that can determine the Pareto set of solutions in a single run, such as those described in [19–22]. Moreover, in some cases, the mathematical programming method and evolutionary optimization algorithm are combined taking advantage of each one to develop a better solution technique [23]. For extensive literature reviews, refer to [24–27].

LINGO [28], one of the most popular optimization software packages, has been extensively used to identify solutions to the problem of optimal operation of reservoir systems. Sharif and Swamy [29] used the Linear Solver and the Branch-and-Bound Solver of LINGO, together with discrete differential dynamic programming (DDDP), to solve the classic hypothetical four-reservoir problem with linear objective function [30] as well as the modified problem with nonlinear objective function [31]. They indicated that the LINGO solvers outperformed DDDP in terms of both solution quality and speed, regardless of whether the objective was formulated with a linear or nonlinear function. Li et al. [32] formulated a mixed-integer linear programming (MILP) model and called the Branch-and-Bound Solver to optimize the hydro unit commitment for the Three Gorges Project in China. The formulation of the MILP model used 14,816 variables (of which 4,608 were binary variables) and 13,329 constraints. In an acceptable computation time, the objective solution was at least within 2.30%-4.11% of the global optimal solution, indicating the powerful performance of LINGO when solving large-scale, mixed-integer, combinatorial optimization problems. Arunkumar and Jothiprakash [33] formulated a model for maximizing the energy generation of a reservoir and used the Global Solver of LINGO to optimize and analyze scenario combinations of various hydrologic years with various constraints. The number of decision variables in this problem is small (tens of decision variables). However, the Global Solver cannot be used to solve high-dimensional nonlinear problems within an acceptable computation time. Salami and Sule [34] formulated the linear model for energy production maximization for a real-world hydropower system and solved several scenarios by using the Linear Solver of LINGO to improve the operating rules. Alemu et al. [35] presented a decision support system that incorporated a simulation model and an optimization model for reservoir system operation. In the system, the optimization model was formulated in LINGO with linear expressions and communicates data using the spreadsheet environment of Microsoft Excel. Undoubtedly, linear formulations have several merits. For instance, such models are easy to solve, and the solutions can be guaranteed to represent global optima. However, real-world problems may be better described with nonlinear formulations without introducing additional variables (particularly integer variables). Furthermore, unlike researchers, operators have considerable management experience, but they may lack mathematical modeling experience. Making use of the powerful performance of optimization software such as LINGO, which is associated with large-scale data management systems, and providing an integrated solution may be the best choice.

This paper presents a LINGO-based integrated framework to study the trade-off between power quantity (maximum energy production) and power reliability (firm output) of the UYR hydropower system, where the two competitive objective functions are major concerns for both power companies and grids. In general, the objective of maximizing energy production lead to the energy production processes to be fluctuated (because it has to adapt to the time-varying inflows); while the objective of improving firm output requires the processes to be stabilized [36–38]. The contributions of this paper can be summarized in the following points. 1) In this study, three layers (the user layer, the coordination layer, and the base layer) are integrated into a framework that is easy to use for operators with little experience in mathematical modeling, takes advantage of the powerful capabilities of LINGO (such as its solving capacity and multi-threading ability), and utilizes a database system for data access and scenario management. The framework can be considered as a simple decision support system (DSS), which can be easily transferred to other hydropower systems with minimal effort and can be extended as the base layer is enriched. 2) A multi-objective model is formulated for the UYR hydropower system to balance maximum energy production and firm output. The model can capture actual characteristics of the UYR hydropower system operation for simulation, and the problem can be solved by LINGO with high solution speed and quality for optimization. 3) With this framework, the operation of the UYR hydropower system is evaluated and optimized during 2000–2010. The energy production potential of the system is explored, and moreover, the trade-off between maximum energy production and firm output is provided for the use of decision makers. The paper proceeds as follows. Section 2 describes the materials used, including the main characteristics of the UYR hydropower system, the hydrological characteristics of the system, and the operating rules of the major reservoirs. Section 3 describes the LINGO-based integrated framework and formulates the multi-objective optimization model for the UYR hydropower system. Section 4 presents and discusses the results obtained. Finally, Section 5 concludes the paper.

## Materials

### UYR hydropower system

**Fig 1:**
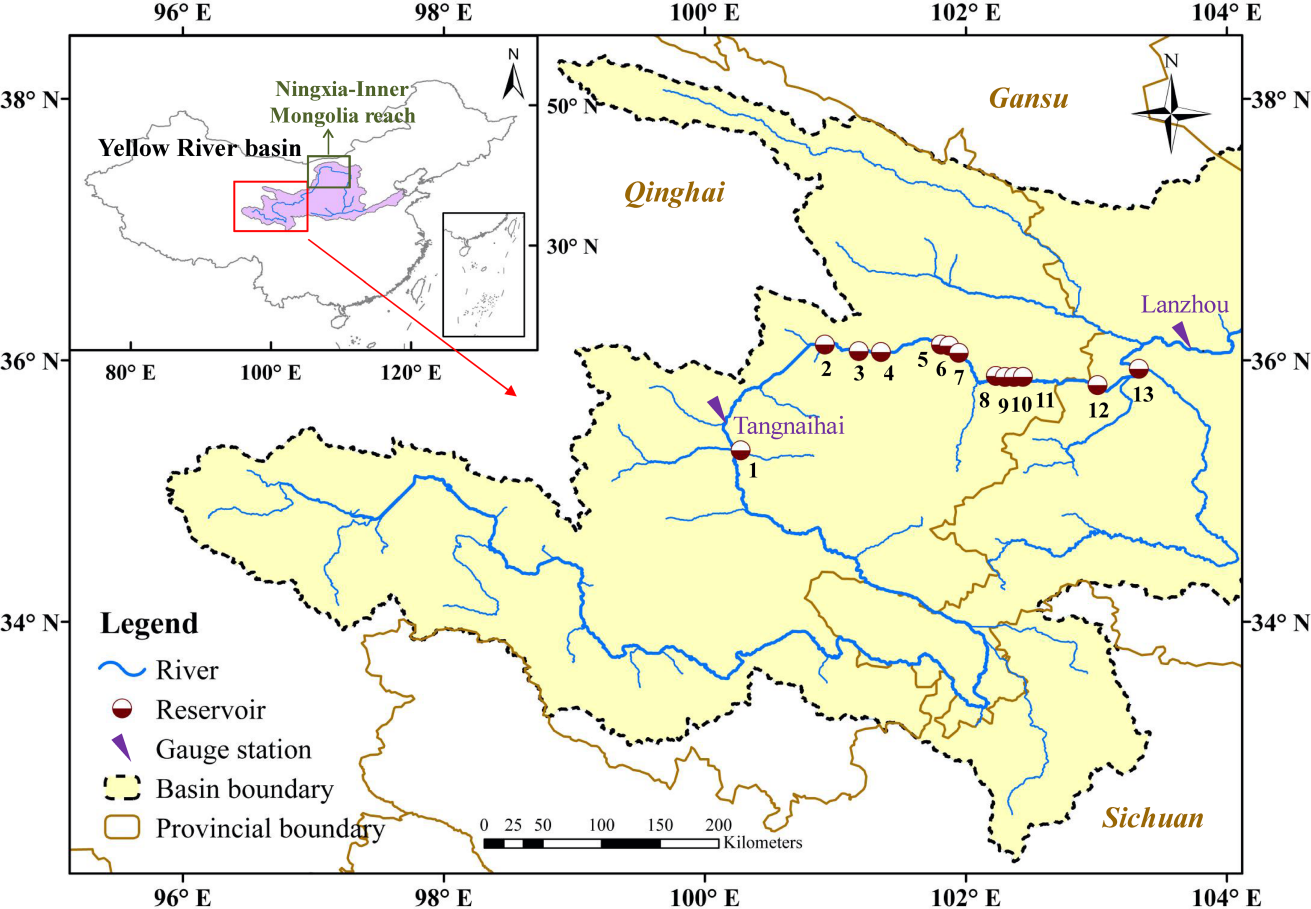
Map of the study area. (Data extracted from the National Geomatics Center of China).

The reservoirs that supply energy to the Qinghai Power Grid are considered (Note that the majority of the installed capacity of the UYR hydropower system is located in Qinghai Province). Along the flow direction, these are the Banduo, Longyangxia (LYX), Laxiwa, Nina, Lijiaxia, Zhiganglaka, Kangyang, Gongboxia, Suzhi, Huangfeng, Jishixia, and Dahejia reservoirs, as shown in Fig 1. The Liujiaxia (LJX) reservoir, although not a constituent of the power grid, is also included due to its large regulation capacity and great significance for the middle and lower basins. It provides benefits such as water resources, ecological flow, and disaster alleviation (e.g., zero-flow prevention and ice/flood control). It should be noted that there are other reservoirs on the UYR downstream of the LJX reservoir that are not considered because they are not part of the power grid and have relatively small regulation capacities. Therefore, a total of 13 reservoirs currently in operation are included. The main characteristics of the reservoirs on the UYR are listed in Table 1.

**Table 1:** Main characteristics of reservoirs on the UYR.

No.	Reservoir	Abbr.	Normal water level (m)	Dead water level (m)	Design water head (m)	Total storage (10^8^ m^3^)	Installed capacity (MW)
**1**	Banduo	BD	2760	2757	35.5	0.108	360
**2**	Longyangxia	LYX	2600	2530	122	247	1280
**3**	Laxiwa	LXW	2452	2440	205	10.79	4200
**4**	Nina	NN	2235.5	2231	14	0.262	160
**5**	Lijiaxia	LIJX	2180	2178	122	16.5	2000
**6**	Zhiganglaka	ZGLK	2050	2048	12.5	0.154	192
**7**	Kangyang	KY	2033	2031	18.7	0.288	280
**8**	Gongboxia	GBX	2005	2002	99.3	5.50	1500
**9**	Suzhi	SZ	1900	1897.5	16	0.455	225
**10**	Huangfeng	HF	1880.5	1878.5	16	0.59	220
**11**	Jishixia	JSX	1856	1852	73	2.38	1020
**12**	Dahejia	DHJ	1783	1782	9.2	0.039	142
**13**	Liujiaxia	LJX	1735	1694	100	57	1350

Among the 13 reservoirs, the LYX and LJX reservoirs are more dominant than other reservoirs because of their large regulation capacities. Reasonable compensation actions between them can improve comprehensive benefits of the operation of the UYR hydropower system. The LYX is a multi-year storage reservoir with 247×10^8^ m^3^ of total storage capacity and 1280 MW of total installed capacity; the LJX is a yearly storage reservoir with 57×10^8^ m^3^ of total storage capacity and 1350 MW of total installed capacity; the others are daily storage reservoirs or run-of-river hydropower plants. The total installed capacity of the 13-reservoir system (including the LJX reservoir) is 12,929 MW, and that of the 12-reservoir system (excluding the LJX reservoir) is 11,579 MW.

### Hydrological characteristics

**Fig 2:**
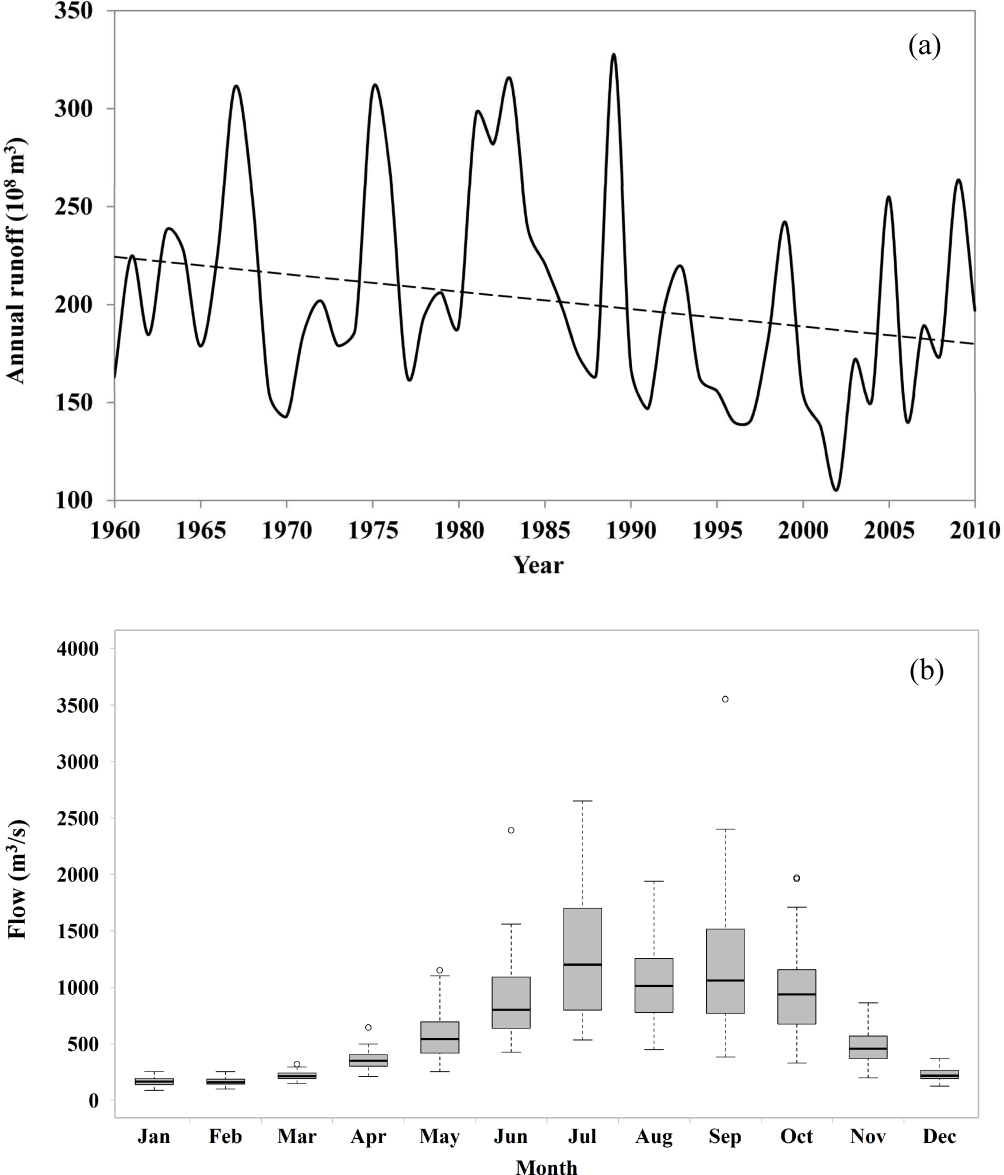
Hydrological characteristics at the Tangnaihai gauge station: (a) the trend of annual runoff; (b) the distribution of monthly flow.

Fig 2 represents the hydrological characteristics from 1960 to 2010 at the Tangnaihai gauge station, which is the control station at the entrance of the LYX reservoir (see Fig 1). The annual average runoff is 202×10^8^ m^3^. As seen from Fig 2(A), the annual runoff has declined over the past 50 years with an average reduction of 0.89×10^8^ m^3^ per year; the maximum annual runoff could reach 327×10^8^ m^3^ before the 1990s, whereas the minimum annual runoff was only 105×10^8^ m^3^ after the 2000s. The UYR region experiences a typical monsoon climate, with heavy precipitation in summer and less in other seasons. The flow results from rainfall during the flood season; it is also fed by flow recession and the melting of mountain snowpack during the non-flood season. As seen from Fig 2(B), the maximum flows occur in July, August, and September, when the annual average flows are 1307, 1078, and 1214 m^3^/s, respectively. On the other hand, the minimum flows occur in December, January, February and March, when the annual average flows are 229, 168, 166, and 220 m^3^/s, respectively. Moreover, the flow variations display a much wider range during the flood season than during the non-flood season.

### Operating rules of major reservoirs

**Fig 3:**
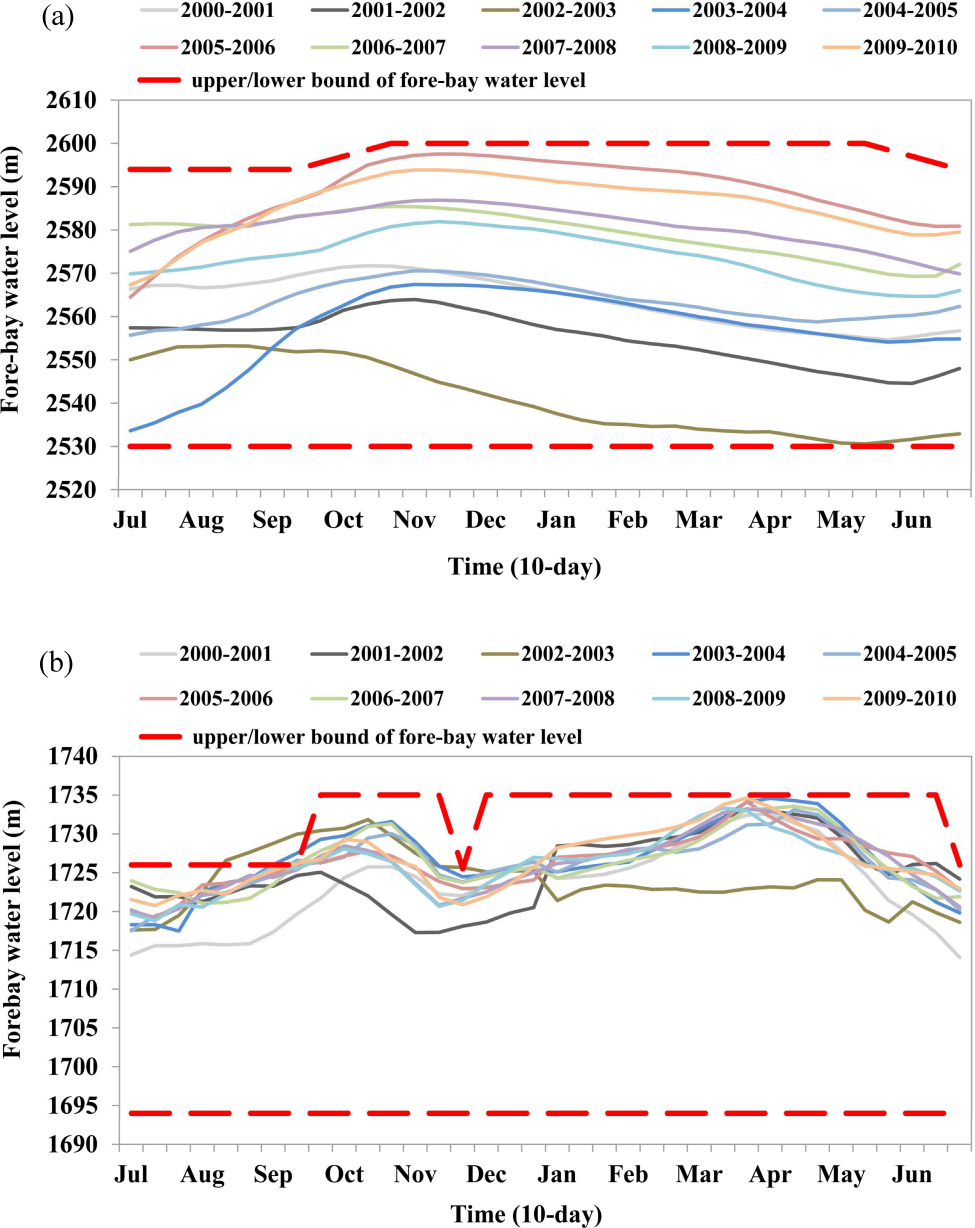
Historical fore-bay water levels of the LYX and LJX reservoirs: (a) the LYX reservoir; (b) the LJX reservoir.

According to the operating rules and practice, the fore-bay water level of the LYX reservoir should be lowered below the flood limited water level of 2594 m before early July (i.e., the beginning of the flood season), and this level should be maintained until the middle of September (i.e., the end of the flood season). Then, the fore-bay water level of the LYX reservoir gradually increases, reaching levels as high as its normal water level of 2600 m (because of its large storage capacity and the limited incoming flow, the LYX reservoir rarely reaches its normal water level in practice). The historical fore-bay water level of the LYX reservoir is shown in Fig 3 (A). The dashed red lines are the upper and lower bounds of the fore-bay water levels, and the other colored solid lines are historical fore-bay water levels from July 2000 to June 2010. The LYX reservoir, as the uppermost multi-year storage reservoir on the Yellow River, controls the allocation of water resources to the whole Yellow River basin; therefore, releases from this reservoir should consider many aspects of water resources utilization, such as hydropower generation, water supply, sediment transport, and ecological flow.

The LJX reservoir undergoes two drawdown-refill cycles during a year, as shown in Fig 3 (B). One is for flood control; the fore-bay water level of the LJX reservoir should be lowered below the flood limited water level of 1726 m before early July, and the impounded water level should be as high as possible after the middle of September. The other is for ice control; the LJX reservoir should be pre-released at the end of November to reserve sufficient storage (approximately 10–15 billion m^3^) because, during the ice season, the releases from the LJX reservoir should be strictly limited to ensure the stability of ice development and breakup, and more water from the LYX reservoir will be released to compensate for the energy deficit during the peak energy period. Note that the LJX is the closest reservoir with regulation capacity to the Ningxia-Mongolia reach (see Fig 1), and it is responsible for preventing ice disasters along this reach, which suffers from severe ice jam or ice dam events due to the flow direction (from low to high latitude) and channel topography every year from November to March of the following year. At times other than these two seasons, the fore-bay water level of the LJX reservoir can reach levels as high as its normal water level of 1735 m.

Furthermore, according to the water institutions, the priority of water regulation is superior to that of hydropower regulation along the Yellow River. Therefore, the releases from the control reservoirs should be guaranteed in order to meet the multiple utilization requirements downstream. The LJX reservoir is currently the storage reservoir of the UYR hydropower system that is farthest downstream, and it should be regarded as the control reservoir. The upper and lower bounds on releases from the LJX reservoir vary among different parts of the year. 1) During the flood season, the upper bound is approximately 4290 m^3^/s, corresponding to the 100-year return period flood at Lanzhou gauge station. 2) During the ice season, the upper bounds range from 400 to 600 m^3^/s, and the lower bounds range from 220 to 400 m^3^/s, according to both practice and the literature [39]. 3) During the water supply season, the lower bounds range from 700 to 950 m^3^/s, particularly for farm irrigation (from April to September), corresponding to the water requirements during 2010 at Lanzhou gauge station [11]. It should be noted that the control releases from the LJX reservoir are presented by converting the flows at Lanzhou gauge station using the basin area ratio for the two locations.

## Methods

### Integrated framework

**Fig 4:**
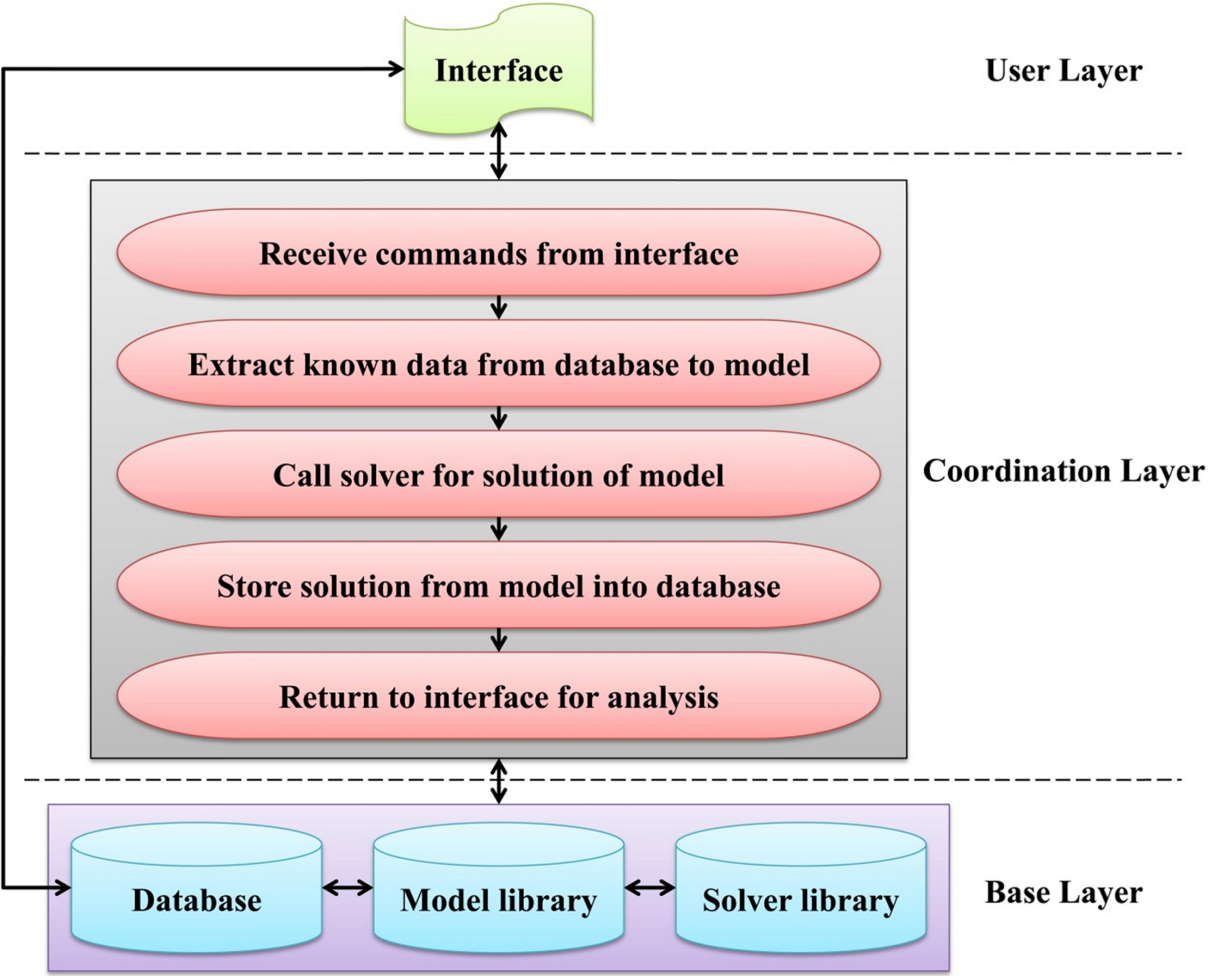
LINGO-based integrated framework for hydropower system simulation and optimization.

Fig 4 shows the LINGO-based integrated framework for hydropower system simulation and optimization. The framework includes three layers, namely, the user layer, the coordination layer, and the base layer.

(1) The user layer, or the user interface, facilitates the use of the framework by operators with little experience in mathematical modeling. It submits commands to the coordination layer and obtains data and analyzes solutions from a database. For simulating or optimizing the operation of a hydropower system, 1) the user first selects a series of settings (or commands) through the user interface, which are stored in the database and flagged by a scenario code for identification. 2) The user interface then passes the scenario code to the coordination layer so that it can locate all the settings. 3) Finally, the user interface receives the message that the simulation or optimization is finished and extracts the solution from database for analysis.

(2) The coordination layer is an application platform, and it is referred to as the “nerve center” of this framework. The major procedures involved in this layer are 1) receiving the scenario code from the user interface and locating the settings in the database for interpretation; 2) extracting the known input data from the database to the computer’s memory and passing them to a model from the model library with the @POINTER function (which is a LINGO function that permits the transfer data through shared memory locations between the application platform and the LINGO software); 3) solving the model by calling an appropriate solver from the solver library using command-line commands (Table 2 shows the scripts for selecting solvers and choosing settings from LINGO. Here, the three commands are pre-defined so that the General Solver, Global Solver, and Multi-start Solver will be called, respectively. The options for multi-thread computing and the number of threads and starting points are also illustrated); 4) passing the solution from the model back to the application platform with the @POINTER function and storing them in the database; 5) finishing the process and returning to the user interface to extract the solution from the database for analysis. The application platform can be compiled into a DLL file that will be called by the user interface using the callable extern function, and the scenario code is a parameter of this function that is used to make connections between the user interface, the application platform, and the database.

**Table 2:** Scripts for selecting solvers and making settings from LINGO.

No.	Scripts	Explanation
**1**	TAKE MODEL.lng SET GLOBAL 0 SET MULTIS 0 GO QUIT	Selects the General Solver to solve the model stored in MODEL.lng
**2**	TAKE MODEL.lng SET GLOBAL 1 SET MULTIS 0 SET MTMODE 1 SET NTHRDS NT SET TIMLIM 3600 GO QUIT	Selects the Global Solver (the multi-thread mode is used, and the number of threads is NT) to solve the model stored in MODEL.lng and terminates the process after 3600 s
**3**	TAKE MODEL.lng SET GLOBAL 0 SET MULTIS NS SET MTMODE 1 SET NTHRDS NT GO QUIT	Selects the Multi-start Solver (the number of starting points is NS; the multi-thread mode is used, and the number of threads is NT) to solve the model stored in MODEL.lng

(3) The base layer includes the database, model library, and solver library, which supports the utility of the integrated framework. The database includes the hydrological data, the physical characteristics of reservoirs, the limitations for reservoir operation, and the boundary conditions, as well as the scenario management data. The database can be updated through networks to access the databases of power companies and grids. The application platform can dynamically connect to the database with the ActiveX Data Object (ADO), which comprises a set of Component Object Model (COM) objects for accessing database sources. In the past few years, the authors have undertaken a series of projects involving reservoir system optimization, such as those of the Three Gorges Project and the UYR hydropower system, and have formulated and accumulated several LINGO models, either for various single objectives or multiple objective trade-offs [17, 32]. Such LINGO models constitute the model library, which will be enriched constantly. The LINGO models are written and stored in LINGO.lng files. The solver library is embedded in the LINGO software, which is capable of solving most classes of optimization models efficiently. The linkage between the application platform and the LINGO software takes place through the LINGO DLL interface. In this study, three NLP solvers are selected for use. These solvers are the General Solver (which uses a generalized reduced gradient-based algorithm), the Global Solver, and the Multi-start Solver. Typically, the General Solver will stop at the first locally optimal solution it identifies, and the outcome is highly dependent upon the starting points. The Global Solver will run for a very long time and search until it confirms it has found the globally optimal solution. Finally, the Multi-start Solver intelligently selects a few different starting points and solves each to a locally optimal solution and then returns the best local optimum found. Furthermore, either serial or parallel computing (i.e., multi-thread computing) can be triggered by the command-line command if the computing environment permits it.

The framework takes full advantages of LINGO’s powerful optimization performance and packs the three layers together into an integrated solution, which is more robust and efficient and is an effective tool for data/scenario management and analysis. The framework is general; it can be easily transferred to other hydropower systems with minimal effort, and it can be extended as the base layer is enriched. Application of this framework can be realized using either the C/S or the B/S structure, where the user layer works on the client or browser, and the coordination layer and base layer work together on the server. In this study, the application platform (i.e., the coordination layer) was developed using the C++ language within the Microsoft Visual Studio 2010 environment, the Microsoft Access 2010 serves as the database for data access, and the 64-bit LINGO software used in this study is the latest release (version 16.0).

### Multi-objective model

For a typical two-objective maximization problem, the mathematical model can be formulated as:

$$\max\;F(x)=\left[F_{1}(x),F_{2}(x)\right]$$ (1)

$$x_{l}\geq 0\;\forall l$$ (2)

$$g_{k}(x)\leq 0\;\forall k$$ (3)

where **F**(**x**) is the vector of objectives that includes two objectives, *F*_1_(**x**) and *F*_2_(**x**); *x*_*l*_ represents the decision variables, *l* ∈ [1,*L*]; *g*_*k*_(**x**) is the constraint, *k* ∈ [1,*K*]. The model can be transformed with the epsilon constraint method into its equivalent formulations, (4)–(7):

$$\max F_{1}(x)$$ (4)

$$F_{2}(x)\geq F_{2}^{\min}+\sigma \cdot \Delta$$ (5)

$$x_{l}\geq 0\;\forall l$$ (6)

$$g_{k}(x)\leq 0\;\forall k$$ (7)

where $F_{2}^{\min}$ is the minimum of objective *F*_2_(**x**); Δ is the increment of *F*_2_(**x**); and *σ* is an integer constant, *σ* = 0,1,2,⋯. The Pareto set of solutions of the two-objective maximization problem can be gained through solving (4)–(7) with various values of *σ*.

### Objective

The objective is to trade off the power quantity and power reliability associated with operation of the UYR hydropower system. To compute the maximum energy production, the energy produced from the LJX reservoir cannot be ignored because of its importance, as mentioned earlier. On the other hand, for computing the firm output, the output production of the LJX reservoir is excluded, because its output production is not transmitted to the Qinghai Power Grid. Therefore, the two optimization objectives can be formulated as (8) and (9):

$$\max\sum_{t=1}^{T}\sum_{i=1}^{13}E_{i}(t)$$ (8)

$$\max\left\{\underset{t}{\min}\left\{\sum_{i=1}^{12}N_{i}(t)\right\}\right\}$$ (9)

The equivalent transformations are:

$$\max\left\{\sum_{t=1}^{T}\sum_{i=1}^{13}E_{i}(t)-\zeta \cdot \sum_{t=1}^{T}\beta (t)\right\}$$ (10)

$$\sum_{i=1}^{12}N_{i}(t)+\beta (t)\geq N^{\min}+\sigma \cdot \Delta N\;\forall t$$ (11)

where

$$E_{i}(t)=N_{i}(t)\cdot \Delta t=9.81\cdot \eta_{i}\cdot R_{i}^{\prime }(t)\cdot H_{i}(t)\cdot \Delta t\;\forall i,\forall t$$ (12)

$$R_{i}(t)=R_{i}^{\prime }(t)+R_{i}^{\prime \prime }(t)\;\forall i,\forall t$$ (13)

$$H_{i}(t)=HF_{i}(t)-HT_{i}(t)-HL_{i}(t)\;\forall i,\forall t$$ (14)

$$HF_{i}(t)=a_{0,i}+a_{1,i}\cdot {\bar{S}}_{i}(t)+a_{2,i}\cdot {\bar{S}}_{i}^{2}(t)\;\forall i,\forall t$$ (15)

$${\bar{S}}_{i}(t)=\left[S_{i}(t)+S_{i}(t-1)\right]/2\;\forall i,\forall t$$ (16)

$$HT_{i}(t)=b_{0,i}\;\forall i,\forall t$$ (17)

*E*_*i*_(*t*) is the energy production of the hydropower plant at reservoir *i* during time step *t*; *N*_*i*_(*t*) is the output production of the hydropower plant at reservoir *i* during time step *t*; *η*_*i*_ is the hydropower plant efficiency at reservoir *i*; $R_{i}^{\prime }(t)$ and $R_{i}^{\prime \prime }(t)$ are the power release and non-power release from reservoir *i* during time step *t*; Δ*t* is the time interval; *H*_*i*_(*t*) is the average head at reservoir *i* during time step *t*, and it represents the difference among the average reservoir fore-bay water level *HF*_*i*_(*t*) (which is a function of the reservoir storage at the beginning and end of the time period) and the average tail-race water level *HT*_*i*_(*t*) (which is set as the elevation of power generating unit for simplicity) and the average water head loss *HL*_*i*_(*t*) at reservoir *i* during time step *t*; *N*^min^ is the minimum output production of the 12-reservoir system; Δ*N* is the increment of output production of the 12-reservoir system; *β*(*t*) is the slack variable during time step *t*; and *ζ* is the penalty factor. With the penalty term in (10) and the slack variable in (11), the model can identify solutions, even if the system output production cannot completely meet the limitation in (11) (i.e., the reliability of firm output is less than 100%).

### Constraints

The operation of the hydropower system is subject to the following constraints:

(1) Continuity equation

S(t+1)=S(t)+I(t)−M⋅R(t)∀t (18)

where **I**(*t*) is the vector of inflows to reservoirs (*i* = 1,⋯,*n*) during time step *t*, **R**(*t*) is the vector of total releases from reservoirs (*i* = 1,⋯,*n*) during time step *t*, **R**(*t*) = [*R*_1_(*t*),⋯,*R*_*i*_(*t*)⋯,*R*_*n*_(*t*)]^*T*^, and **M** is the *n*×*n* reservoir system connectivity matrix. Without loss of generality, we assume that evaporation loss is balanced by precipitation.

(2) Initial and final reservoir storage

$$S(1)=S^{initial}$$ (19)

$$S(T+1)\geq S^{final}$$ (20)

where **S**^*initial*^ and **S**^*final*^ are vectors containing the initial and final amounts of water stored in reservoirs (*i* = 1,⋯,*n*).

(3) Lower and upper bounds on storage

$$S^{\min}(t+1)\leq S(t+1)\leq S^{\max}(t+1)\;\forall t$$ (21)

where **S**^min^(*t*+1) and **S**^max^(*t*+1) are the vectors of the minimum and maximum storage of reservoirs (*i* = 1,⋯,*n*) at the end of time step *t*.

(4) Lower and upper bounds on total releases

$$R^{\min}(t)\leq R(t)\leq R^{\max}(t)\;\forall t$$ (22)

where **R**^min^(*t*) is the vector of the minimum required releases from reservoirs (*i* = 1,⋯,*n*) during time step *t*, and **R**^max^(*t*) is the vector of maximum allowable releases from reservoirs (*i* = 1,⋯,*n*) during time step *t*.

(5) Lower and upper bounds on power releases

$$R^{\prime \min}(t)\leq R^{\prime }(t)\leq R^{\prime \max}(t)\;\forall t$$ (23)

where **R**′^min^(*t*) is the vector of the minimum required releases through the turbines in hydropower plants at reservoirs (*i* = 1,⋯,*n*) during time step *t*; and **R**′^max^(*t*) is the vector of the maximum allowable releases through turbines in hydropower plants at reservoirs (*i* = 1,⋯,*n*) during time step *t*.

(6) Lower and upper bounds on outputs

$$N^{\min}(t)\leq N(t)\leq N^{\max}(t)\;\forall t$$ (24)

where **N**(*t*) is the vector of outputs produced from hydropower plants at reservoirs (*i* = 1,⋯,*n*) during time step *t*, **N**(*t*) = [*N*_1_(*t*),⋯,*N*_*i*_(*t*)⋯,*N*_*n*_(*t*)]^*T*^, **N**^min^(*t*) is the vector of minimum required outputs from hydropower plants at reservoirs (*i* = 1,⋯,*n*) during time step *t*, and **N**^max^(*t*) is the vector of maximum allowable outputs from hydropower plants at reservoirs (*i* = 1,⋯,*n*) during time period *t*.

Note that releases are decision variables. The other state variables can be calculated once the decision variables are determined. The inflows, the initial and final conditions, as well as the limitations on reservoir operation are the known input data in the optimization.

## Case study

The inflows over a recent 10-year period that extends from July 2000 to June 2010 are used as inputs to the formulated model. The time period is divided into 360 time steps (each of which has a length of approximately 10 days). The annual runoff and its hydrologic frequency during 2000–2010 at the Tangnaihai gauge station are shown in Table 3, where the hydrologic year is used (e.g. 2000–2001 represents July 2000-June 2001). In practice, the water supply requirement downstream of the LJX reservoir cannot be adequately met during dry years. Therefore, it is assumed that the releases from the LJX reservoir can reach 80% of the required values during the water supply seasons for the years when the hydrologic frequencies are above 80% (i.e. 2000–2001, 2001–2002, 2002–2003, and 2006–2007). For analysis, three typical hydrologic years, including a dry year (2004–2005), a normal year (2008–2009) and a wet year (2005–2006), are selected. These hydrologic years correspond to the hydrologic frequencies of 71%, 45% and 22% in the historical record (1960–2010), respectively.

**Table 3:** Annual runoff and its hydrologic frequency during 2000–2010 at the Tangnaihai gauge station.

Year	2000 -2001	2001 -2002	2002 -2003	2003 -2004	2004 -2005	2005 -2006	2006 -2007	2007 -2008	2008 -2009	2009 -2010
**Runoff** **(10**^**8**^ **m**^**3**^**)**	146.2	130.7	97.1	176.2	167.2	245.4	151.0	173.0	204.3	251.3
**Frequency** **(%)**	86	92	99	67	71	22	82	66	45	19

The simulation is performed first, followed by the optimization. For both the simulation and optimization, the models are same, except that the releases from the reservoirs are known in the simulations and are to be optimized in the optimizations. The LYX and LJX reservoirs are operated as storage reservoirs, whereas the other reservoirs are treated as run-of-river hydropower plants due to their small regulation capacities. The initial and final reservoir storage values are same as the observed values; that is, the total water amounts released from the reservoirs are same as the observed values.

All computations were performed on a MacBook Pro containing an Intel (R) Core (TM) i7-3520 M CPU with 2.90 GHz and 8.00 GB of RAM.

## Results and discussion

### Simulation

**Fig 5:**
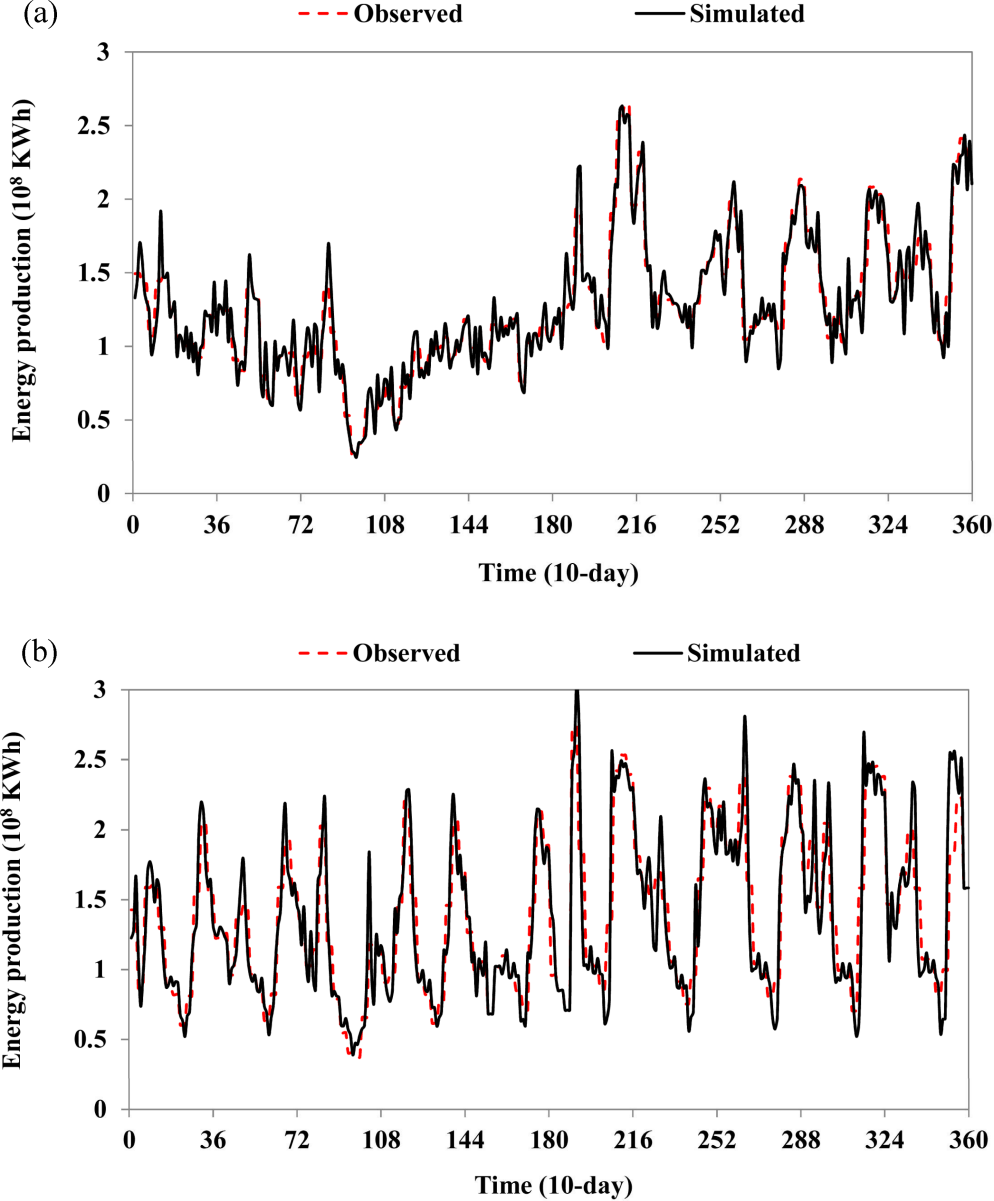
Comparison of observed and simulated energy productions of the LYX and LJX reservoirs: (a) the LYX reservoir; (b) the LJX reservoir.

During 2000–2010, the LYX and LJX reservoirs were in operation, and each reservoir collected a complete set of data. The actual releases from LYX and LJX reservoirs are known, and the releases from other reservoirs can be determined by computation accordingly. For instance, the releases from the BD reservoir (the most upstream reservoir in the system) are equal to the inflows to the reservoir; the releases from the LXW reservoir, directly downstream of the LYX reservoir, are equal to the releases from the LYX reservoir plus the interval flows between the LYX reservoir and the LXW reservoir; other releases from other reservoirs can be gained similarly. Given releases from all 13 reservoirs as well as other known data used in the model, the simulation can be performed on the assumption that all 13 reservoirs are in operation during this period (in fact, some of these reservoirs were not operational or did not collect all of the relevant data). Fig 5 shows comparisons of the observed and simulated power generations of the LYX and LJX reservoirs. It should be noted that the LJX reservoir has to release considerable amounts of water (without producing power) from the turbines of the hydropower plant to reserve capacity for the grid during some periods, when the simulated power generation has been modified accordingly. It can be seen that the simulated results coincide well with the observed values (the coefficients of determination are close to 1.0). The annual average power generation is estimated to be 356.8×10^8^ KWh for the 13-reservoir system and 306.0×10^8^ KWh for the 12-reservoir system, with power generation values of 267.7, 357.5, and 358.3×10^8^ KWh for the dry, normal, and wet years, respectively. The results indicate the following conclusions. 1) The parameters selected for use within the formulated model are reasonable, and the model can be used for evaluation and optimization of the UYR hydropower system operation; 2) the LINGO-based integrated solution can be used for accurate simulations other than optimizations, and the comprehensive benefits of operation of hydropower systems can be estimated once decision variables (i.e., releases) are given; 3) the simulation can be seen as the baseline scenario (each storage reservoir in the system is operated individually rather than jointly) for getting know the energy production potential by comparison with the joint optimal operation of the system.

### Optimization

**Fig 6:**
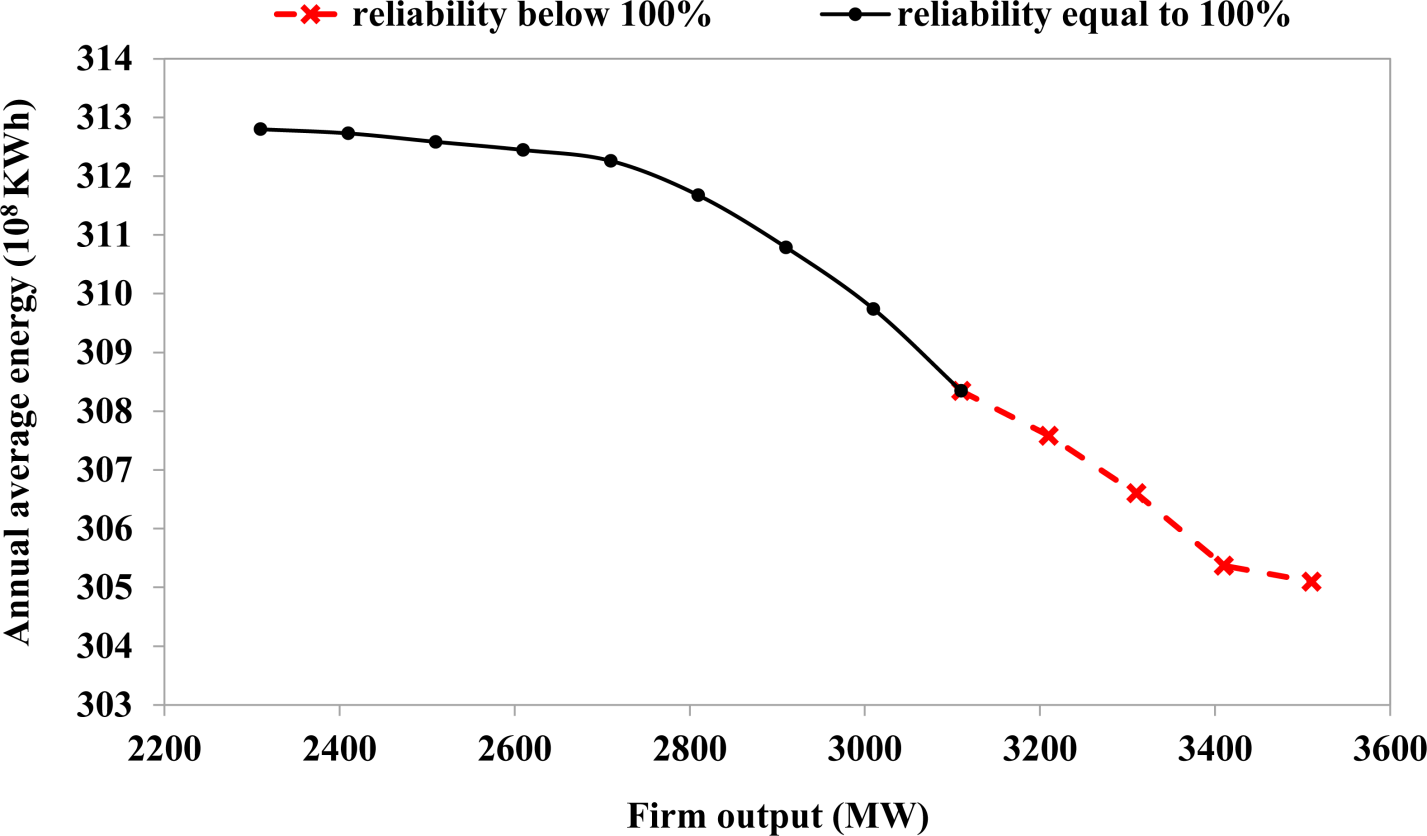
Trade-off between maximum energy production and firm output of 12-reservoir system in the Qinghai Power Grid.

As mentioned previously, the optimization can be performed once all of the data, except for the releases from reservoirs, have been given. The optimization model is highly nonlinear and non-convex because of the complex conversion from potential energy to kinetic energy and then to electrical energy in the objective function [40]. Additionally, because of the large number of reservoirs, as well as the extended time period covered, the computational dimension is extremely high: a total of 51,480 variables (15,480 of which are nonlinear variables) and 42,134 constraints (6120 of which are nonlinear constraints) are involved in the optimization. It is due to these mathematical characteristics that three NLP solvers embedded in LINGO–General Solver, Global Solver, and Multi-start Solver are selected for solution verification. Table 4 shows comparison of various NLP solvers for S1 of Table 5. The General Solver gains the solution fastest with a single thread, and its runtime is 180 s. On the other hand, the Global Solver runs for a very long time, and although 2 threads are used, it could only be confirmed that the maximum objective bound is 3690.03 until the runtime is terminated (~1 hour). The Multi-start Solver searches the same solution with the General Solver, although it employs various numbers of starting points. Generally, the solution quality of the Multi-start Solver will improve as the number of starting points increases. There should be a half dozen or so distinct local optima; however, they are all alternate optima with a same objective value. The coincidence is probably due to some sort of symmetry in the problem. Furthermore, it can witness the obvious decrease in runtime once the option for multi-thread computing is activated. However, several reasons exist that explain why using n threads gives substantially less than n times speed up, such as load balancing, memory congestion, clock speed reduction, and algorithmic inefficiencies. In any case, the reliability of the identified solutions to the large-scale nonlinear hydropower system optimization can be verified through comparing these NLP solvers. The trade-off between the maximum energy production and the firm output of the UYR hydropower system is shown in Table 5, where 13 scenarios (i.e., S1-S13) are listed. Note that, in practice, a much larger number of scenarios should be analyzed in order to develop reliable reservoir operation plans. For comparison, the simulation scenario (i.e., S0) is also listed. Both the annual average energy production of the 13-reservoir system (including the LJX reservoir) and the 12-reservoir system (excluding the LJX reservoir) are presented. It can be seen from S1 that the maximum annual average energy production of the 13-reservoir system is 364.9×10^8^ KWh and that of the 12-reservoir system is 312.8×10^8^ KWh. The annual average energy production decreases as the firm output of the reservoir system increases. The firm output can reach 3110 MW if the reliability is equal to 100%, whereas it can reach 3510 MM if the reliability is approximately 90%. Note that the reliability is the ratio of the number of time steps when the firm outputs are met to the total number of time steps. A small decrease in annual average energy production can bring about a substantial increase in firm output from the 12-reservoir system. Specifically, a 4.5×10^8^-KWh (1.42%) decrease in maximum energy production produces an 800-MW (34.63%) increase in firm output (i.e., S9 minus S1); a 7.7×10^8^ KWh (2.46%) decrease in maximum energy production yields an 1200-MW (51.95%) increase in firm output in return (i.e., S13 minus S1). The decrease in annual average energy production from the 12-reservoir system is completely from the LYX reservoir. Moreover, a comparison between the optimized and simulated outcomes (i.e., S0 vs. S1 and S0 vs. S9), it can be seen that there is room for improvement in the joint operation, particularly in terms of the system’s firm output. The Pareto set of solutions is shown in Fig 6, where a solution indicates the annual average energy (i.e., the maximum total energy production divided by 10 years) corresponding to the set firm output of the UYR hydropower system. Four scenarios (S1, S5, S9, and S13) are selected for use in the following analysis.

**Table 4:** Comparison of various NLP solvers for S1.

Solver settings	General Solver	Global Solver	Multi-start Solver					
		Thread(s) = 2	Starting point(s) = 2, Thread(s) = 1	Starting point(s) = 2, Thread(s) = 2	Starting point(s) = 4, Thread(s) = 1	Starting point(s) = 4, Thread(s) = 2	Starting point(s) = 6, Thread(s) = 2	Starting point(s) = 6, Thread(s) = 3
**Runtime (s)**	180	3600^a^	304	201	994	465	949	1036
**Objective**	3649.02	Objective bound = 3690.03	3649.02	3649.02	3649.02	3649.02	3649.02	3649.02

^a^ The termination time is set to 3600 seconds (1 hour).

**Table 5:** Trade-off between maximum energy production and firm output of UYR reservoir system.

Scenario	Firm output (MW)	Annual average energy production (10^8^ KWh)			
		LYX	LJX	13-reservoir system	12-reservoir system
**S0**	3110 (59.2%)	45.7	50.8	356.8	306.0
**S1**	2310	51.9	52.1	364.9	312.8
**S2**	2410	51.8	52.1	364.8	312.7
**S3**	2510	51.7	52.0	364.6	312.6
**S4**	2610	51.5	51.9	364.4	312.4
**S5**	2710	51.4	51.8	364.1	312.3
**S6**	2810	50.8	51.9	363.6	311.7
**S7**	2910	49.9	52.0	362.8	310.8
**S8**	3010	48.8	52.1	361.8	309.7
**S9**	3110	47.4	51.9	360.3	308.3
**S10**	3210 (97.8%)^a^	46.7	51.9	359.6	307.6
**S11**	3310 (95.6%)	45.7	51.9	358.5	306.6
**S12**	3410 (93.3%)	44.5	51.7	357.1	305.4
**S13**	3510 (90.6%)	44.2	51.2	356.3	305.1

^a^ The percentage after the firm output represents the reliability.

**Fig 7:**
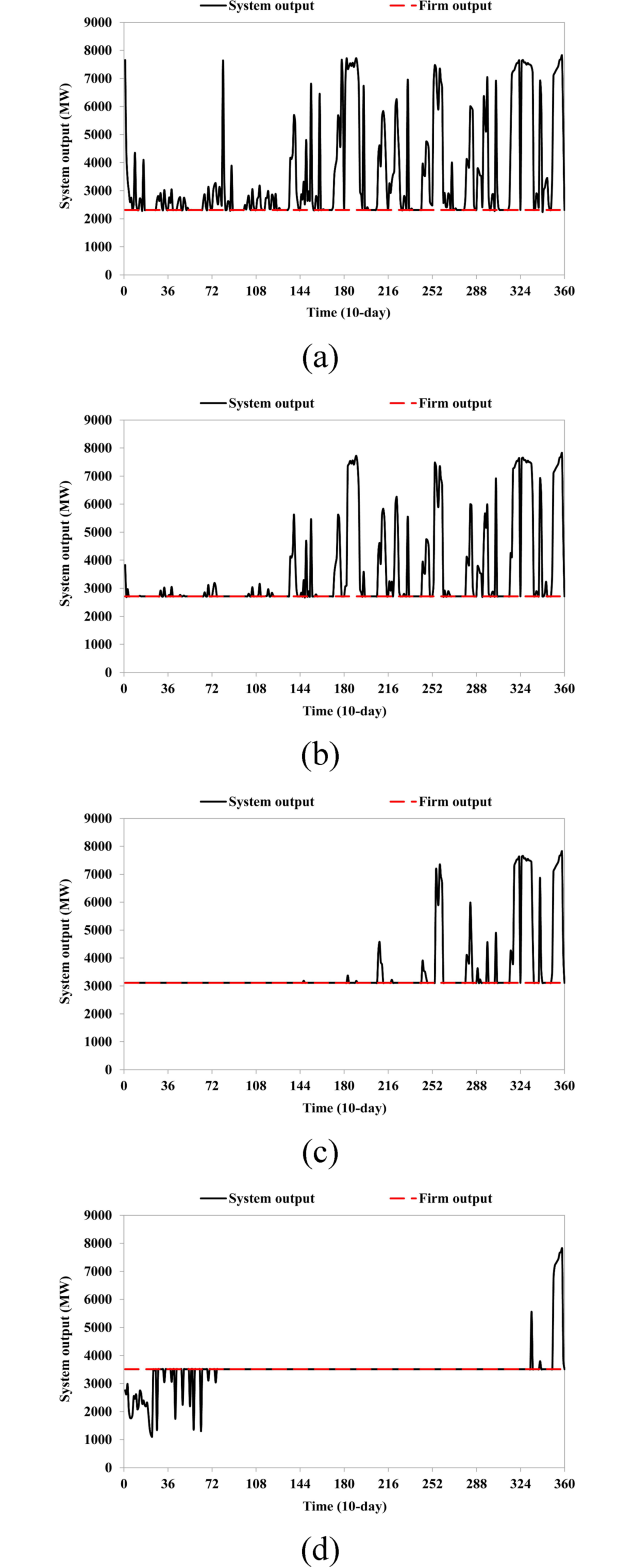
Comparison of output production from 12-reservoir system among various scenarios: (a) S1; (b) S5; (c) S9; (d) S13.

The energy production during various hydrologic years is shown in Table 6. In S1, the maximum energy production of the 12-reservoir system can reach 297.7, 363.9, and 411.4×10^8^ KWh, and the energy production from LYX reservoir is 49.5, 62.8, and 71.1×10^8^ KWh for dry, normal, and wet years, respectively. In S13, the maximum energy production of the 12-reservoir system is 307.5×10^8^ KWh, and the energy generation of the LYX reservoir is 35.9, 46.2, and 44.5×10^8^ KWh for the three typical hydrologic years. This indicates that the LYX reservoir regulates water conditions over multiple years and distributes the water and hydropower resources from wet years to dry years to meet the firm output requirement. A comparison of output production from the 12-reservoir system for the various scenarios is shown in Fig 7. It can be seen that the system output becomes flatter and stable as the firm output increases (because in Fig 7(A)–7(D) the numbers of system outputs equal to the firm outputs are increased). In S1, S5, and S9, the system output can completely meet the firm output requirement. On the other hand, there are some violations in S13 that occur in the first consecutive three driest years from 2000 to 2003. There are several probable reasons for the violations: 1) the system output cannot reach the firm output at the beginning subject to the series of constraints; 2) the total energy production can be maximized during the entire time horizon if the violations occur at the beginning; 3) the number of violations is least if they occur at the beginning.

**Table 6:** Energy production during various hydrologic years.

Hydrologic year	Scenario	Energy production (10^8^ KWh)			
		LYX	LJX	13-reservoir system	12-reservoir system
**Dry**	S0	37.4	46.7	304.3	267.7
	S1	49.5	50.4	348.1	297.7
	S5	44.0	47.3	323.6	276.3
	S9	35.2	45.7	318.3	272.7
	S13	35.9	53.7	361.2	307.5
**Normal**	S0	54.4	58.4	402.5	357.5
	S1	62.8	59.8	423.7	363.9
	S5	62.0	59.2	418.9	359.7
	S9	58.6	56.6	398.7	342.1
	S13	46.2	51.2	358.7	307.5
**Wet**	S0	59.4	61.8	405.2	358.3
	S1	71.1	70.2	481.6	411.4
	S5	68.0	67.8	461.7	393.9
	S9	45.2	52.7	335.6	282.9
	S13	44.5	57.3	364.9	307.5

**Fig 8:**
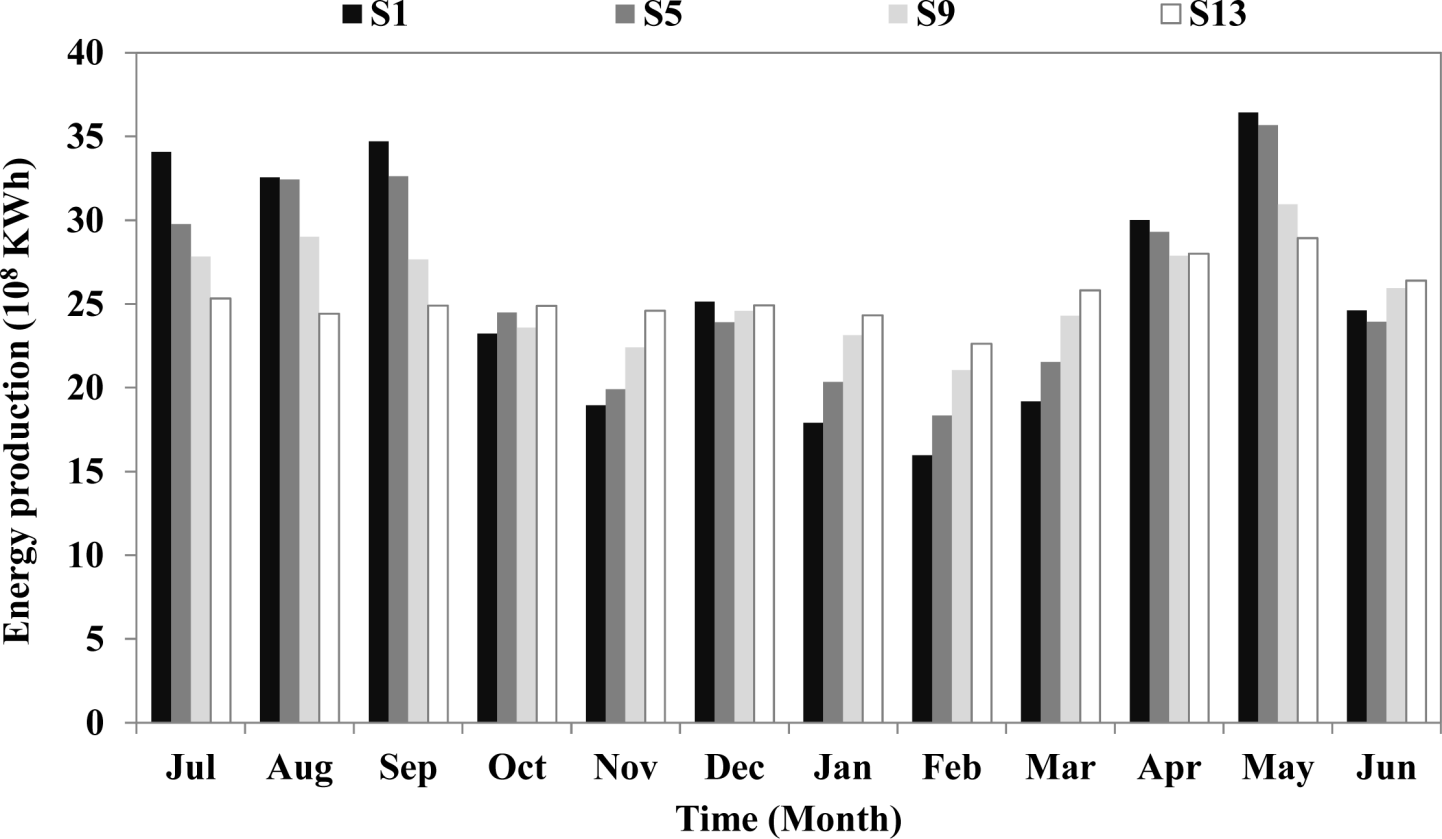
Comparison of energy production distribution from 12-reservoir system among various scenarios.

**Fig 9:**
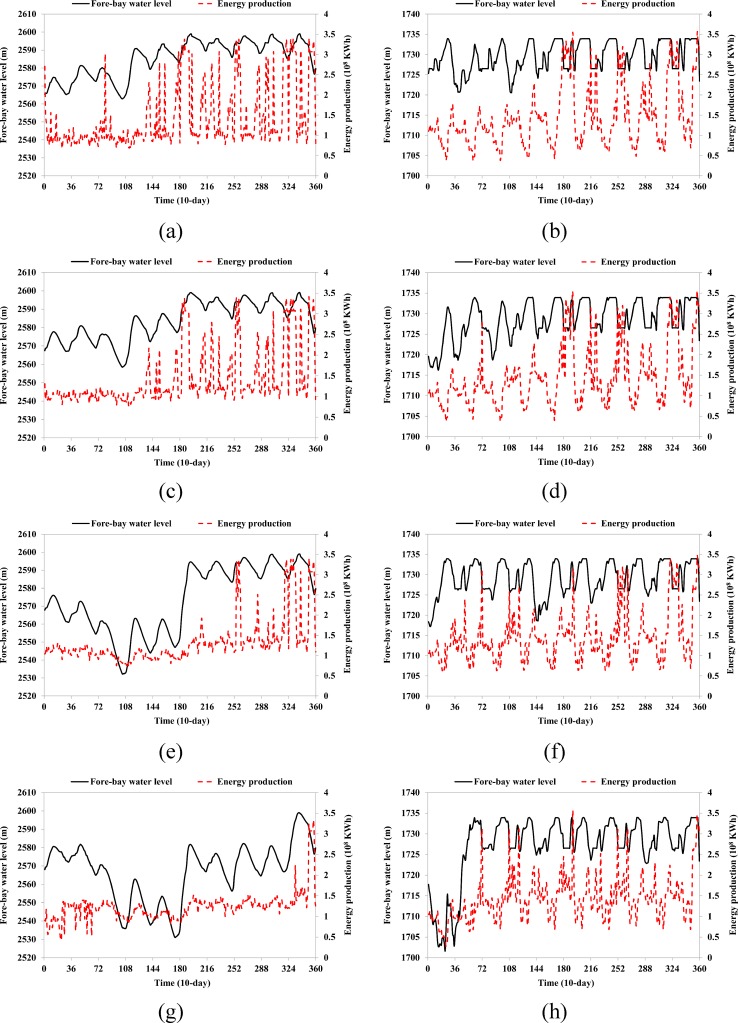
Comparison of fore-bay water level and release among various scenarios: (a) S1; (c) S5; (e) S9; (g) S13 for the LYX reservoir, and (b) S1; (d) S5; (f) S9; (h) S13 for the LJX reservoir.

The comparison of energy production distribution from the 12-reservoir system among various scenarios is shown in Fig 8. To maximize the energy production, the annual average energy production is unevenly distributed among the various months within a year—more energy is produced during the wet season (May, July, August, and September) while less energy is produced during the dry season. In S1, the maximum energy production is 36.4×10^8^ KWh in May, and the minimum energy production is 16.0×10^8^ KWh in February. To maximize the firm output, the annual average energy production is evenly distributed. In S13, the energy production is approximately 25.0×10^8^ KWh among the various months. A comparison of fore-bay water level and release among the various scenarios is shown in Fig 9. It can be seen that, as the firm output increases, the release from the LYX reservoir becomes higher and flatter, resulting in a general decrease in the fore-bay water level of the LYX reservoir. In S13, the water stored in the LYX reservoir plus the incoming flow to the LYX reservoir cannot maintain the increase in the release from the LYX reservoir, resulting in the violations for the firm output from the 12-reservoir system during the first three years. On the other hand, the operation of the LJX reservoir adapts to various incoming flows from upstream in the different scenarios for energy production maximization.

## Conclusions

This paper presents a generalized LINGO-based integrated framework to study the operation of the hydropower system in the Upper Yellow River (UYR), the conclusions of which are summarized below.

The LINGO-based integrated solution can be used for accurate simulation and complex optimization of hydropower system operation. In particular, models that involve nonlinearity, non-convex solution surfaces and high dimensionality can be solved reliably by the LINGO solvers. The solution quality can be verified through comparing the various solvers, and the solution speed can be greatly improved through employing multi-threaded computing. The framework is easy for operators with little experience in mathematical modeling to use, takes full advantage of LINGO’s capability, and packs its three layers (the user layer, the coordination layer, and the base layer) together into an integrated solution. This solution is comparatively robust and efficient, and the framework is an effective tool for data/scenario management and analysis. The framework is general and can be easily transferred to other hydropower systems with minimal effort, and it can be extended as the base layer is enriched.

Both simulation and optimization are performed with the LINGO-based integrated solution to verify the accuracy and to evaluate and optimize the operation of the UYR hydropower system, including the most important reservoirs on the Yellow River, which are the Longyangxia (LYX) Reservoir and the Liujiaxia (LJX) Reservoir. The multi-objective model that represents the trade-off between maximum energy production and firm output has been formulated and is solved by the NLP solvers embedded in LINGO, such as the General Solver, the Multi-start Solver, and the Global Solver. The inflow over a recent 10-year period is provided as input to the model for analysis. The results indicate that 1) the maximum annual average power production can reach 312.8×10^8^ KWh, corresponding to a firm output of 2310 MW. On the other hand, the maximum firm output can reach 3110 MW (reliability of 100%) or 3510 MW (reliability of 90%), corresponding to a maximum annual average power production of 308.3×10^8^ KWh and 305.1×10^8^ KWh, respectively, for the 12-reservoir system (excluding the LJX reservoir); reductions in the maximum energy production by 1.42% and 2.46%, which is completely from the LYX reservoir, can yield increases in firm output of 36.63% and 51.95%, respectively. 2) For typical dry, normal, and wet years, the maximum energy production can reach 297.7, 363.9, and 411.4×10^8^ KWh, respectively. When the firm output is 3510 MW, the energy production is 307.5×10^8^ KWh for all the three hydrologic years for the 12-reservoir system, indicating that the LYX reservoir regulates water conditions over multiple years and distributes the water and hydropower resources from wet years to dry years to meet the firm output requirement. 3) To maximize the energy production, the fore-bay water level of the LYX reservoir should be kept as high as possible. On the other hand, to maximize the firm output, the water from the LYX reservoir should be released as much and stable as possible. 4) A comparison of the simulated and optimized solutions shows that there is potential for improvement in the joint operation of the UYR hydropower system. The Pareto set of solutions, which has the form of a trade-off curve, can provide important information for managers and help support power purchasing decisions from outside Qinghai Province.

Future works could implement the artificial intelligence and data mining techniques to predict the runoff to the UYR hydropower system in the changing hydrological and climatic conditions (so as to enable forecast-informed operations), and to extract and improve the reservoir operation rules for the UYR hydropower system management, as described in [41, 42].

## Supporting information

S1 File Data and results. (XLS)

S2 File Result comparisons between LINGO and GAMS. (DOCX)
