# Supplementary material for: Evaluating and optimizing the operation of the hydropower system in the Upper Yellow River: A general LINGO-based integrated framework
Source: PLoS One. 2018 Jan 25;13(1):e0191483. doi: 10.1371/journal.pone.0191483 (PMC5784971; doi:10.1371/journal.pone.0191483)
Supplement: S2 File — (DOCX) [file pone.0191483.s002.docx]

**S2 File. Result comparisons between LINGO and GAMS.**

The optimization problem in Scenario 1 of this paper (where the firm output of the UYR hydropower system is 2310 MW) was also solved with GAMS 23.3, similar to the model formulation (including the objective function and constraints) and inputs to the model with LINGO 16.0. The two commonly-used NLP solvers, namely CONOPT3 and MINOS5, embedded in GAMS were called for solution. It is worth noting that both solvers from LINGO and GAMS were used with their default settings (although adjustments may produce solutions of better qualities). The comparison of objectives from various solvers for Scenario 1 is listed in Table 1. The solution reports of the two GAMS NLP solvers are shown in Figs 1 and 2. Comparisons of the fore-bay water levels of the Longyangxia Reservoir and the Liujiaxia Reservoir, as well as the system output are shown in Figs 3-5. It is clear to see the reliability of the solution gained by LINGO.

**Table 1. Comparison of objectives from various solvers for Scenario 1.**

| Solver | LINGO  (General Solver) | GAMS  (CONOPT3 Solver) | GAMS  (MINOS5 Solver) |
| --- | --- | --- | --- |
| Objective | 3649.02 | 3644.10 | 2843.47 |


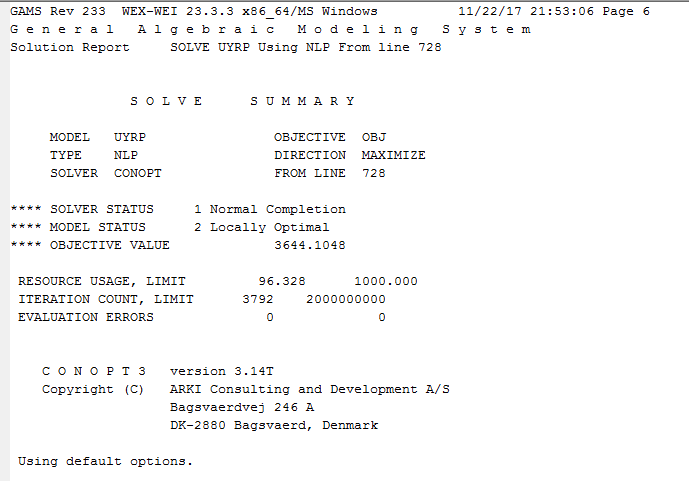


**Fig 1. Solution report using CONOPT3 solver with default settings.**


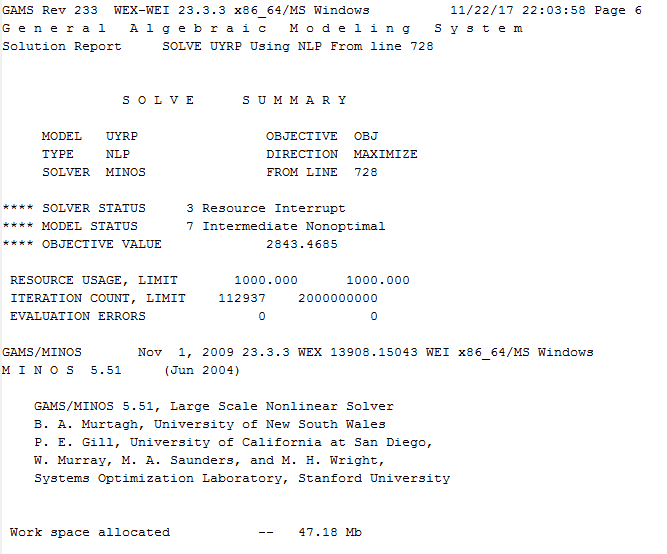


**Fig 2. Solution report using MINOS5 solver with default seetings.**

**Fig 3. Comparison of fore-bay water levels of Longyangxia Reservoir.**

**Fig 4. Comparison of fore-bay water levels of Liujiaxia Reservoir.**

**Fig 5. Comparison of output productions from 12-reservoir system.**
